# Eptesicus fuscus Orthorubulavirus, a Close Relative of Human Parainfluenza Virus 4, Discovered in a Bat in South Dakota

**DOI:** 10.1128/Spectrum.00930-21

**Published:** 2021-10-20

**Authors:** Ben M. Hause, Eric Nelson, Jane Christopher-Hennings

**Affiliations:** a Animal Disease Research and Diagnostic Laboratory, South Dakota State University, Brookings, South Dakota, USA; b Department of Veterinary and Biomedical Sciences, South Dakota State University, Brookings, South Dakota, USA; Labcorp

**Keywords:** bat, parainfluenza virus, paramyxovirus, orthorubulavirus

## Abstract

Bats are a reservoir for many zoonotic viruses and host large numbers of genetically diverse species in the families *Rhabdoviridae*, *Coronaviridae*, and *Paramyxoviridae*. Viruses from these families have repeatedly spilled over to humans in recent decades, causing significant clinical disease and deaths. Here, metagenomic sequencing of a big brown bat (Eptesicus fuscus) submitted for rabies testing due to human exposure identified a novel paramyxovirus, Eptesicus fuscus orthorubulavirus (EfORV), in South Dakota, United States. The nearly complete 15,814-nucleotide genome shared 72% identity with that of human parainfluenza virus 4 (HPIV4), a virus that causes significant clinical disease, typically bronchiolitis and pneumonia, in children less than 2 years of age. Phylogenetic analysis confirmed a close evolutionary history between EfORV and HPIV4, reminiscent of other orthorubulaviruses with highly similar bat and mammalian species, including conspecific human and bat mumps virus, mammalian parainfluenza virus 5 and bat Alston virus, and porcine La Piedad Michoacán virus and bat Mapuera virus. These results support the idea that bats are a reservoir for diverse paramyxoviruses with closely shared evolutionary histories, compared with a number of significant human pathogens, and expand the range of bat paramyxoviruses to North America. Given the propensity of paramyxoviruses to overcome species barriers, additional surveillance and characterization of EfORV are warranted.

**IMPORTANCE** Bats are a reservoir of large numbers of viruses. Among bat-borne zoonotic viruses, members of *Coronaviridae* and *Paramyxoviridae* have had the largest impact on human health. The repeated spillover of bat viruses to humans, often with devastating results, has led to increased surveillance and virus discovery efforts in hot spots for virus emergence, largely Asia and Africa. Apart from rabies virus, little surveillance of viruses in bats is performed in North America. Here, viral metagenomic sequencing identified a close relative to HPIV4 in a big brown bat found in a motel room in South Dakota. The virus, EfORV, was 72% identical to HPIV4, which causes clinically significant respiratory disease, mainly in children; it represents the first bat paramyxovirus identified in North America. Close genetic relationships between bat and mammalian orthorubulaviruses underscore the importance of bats as a reservoir for zoonotic viruses.

## OBSERVATION

Bats are an important reservoir for zoonotic viruses. In the nearly one century since the discovery of rabies virus in bats, a large number of clinically significant viruses have emerged from a bat reservoir (1, 2). Repeated spillovers of coronaviruses, including severe acute respiratory syndrome coronaviruses and Middle East respiratory coronavirus, have had dramatic effects on human and livestock health. Besides coronaviruses, the families *Rhabdoviridae* and *Paramyxoviridae* include bats as a reservoir for numerous species capable of zoonosis.

Several bat-borne paramyxoviruses have been transmitted to humans. Hendra virus and Nipah virus, both of the genus *Henipavirus*, emerged from bats in Australia and Malaysia, respectively (1). Although only a few hundred human cases were diagnosed, case fatality rates were 57% and 39%, respectively. Menangle virus, of the genus *Pararubulavirus*, was identified in pigs with reproductive disease and was transmitted to farm workers, causing significant disease (3). Sosuga virus, another pararubulavirus, was found concurrently in bats and in a wildlife researcher with febrile illness (4).

Targeted detection of paramyxoviruses using PCR with conserved primers in specimens from 119 bat and rodent species collected from 15 sites worldwide found 66 novel paramyxoviruses based on partial L gene sequencing (5). Phylogenetic analyses found that bats are the most common reservoir host for paramyxoviruses transmitted to other species. Close phylogenetic relationships were seen between bat paramyxoviruses and significant human paramyxoviruses, including mumps virus, rubella virus, and human parainfluenza viruses (HPIVs).

Despite the large amount of bat surveillance performed worldwide, comparably little surveillance has been performed in North America. In an effort to detect possible zoonotic viruses in synanthropic bats, metagenomic sequencing was performed on a tissue homogenate prepared from the viscera of a bat submitted for rabies testing. Direct fluorescent antibody testing of the brain yielded negative results for rabies virus. The bat was identified in a motel in central South Dakota and reportedly was crawling on the floor. A total of 852,138 paired-end 151-bp reads were used to assemble a 15,814-bp contig with 72.3% similarity to the orthorubulavirus HPIV4. The virus was named Eptesicus fuscus orthorubulavirus (EfORV) strain 6931. To our knowledge, this is the first bat paramyxovirus identified in North America.

Open reading frame (ORF) analysis identified canonical paramyxovirus genome organization. The partial N gene, encoding 551 amino acids (aa), was incomplete, missing approximately 6 aa based on sequence alignment with HPIV4b N, which was 74.9% identical. The nonstructural V gene was the second ORF and encodes a 218-aa protein with 37.3% identity to that of HPIV4b. The P gene, encoding a 391-aa protein with 42.7% identity to that of HPIV4b, shares its amino terminus with V, with a predicted RNA editing site of 5′-AAGAGGGGG-3′ (mRNA sense) located at nucleotide positions 2393 to 2401 leading to insertion of two nontemplated G residues and continuation of translation in an alternate reading frame. The remaining ORFs, for the M, F, HN, and L genes, code for 381, 544, 586, and 2,273 aa, respectively, which are 71.4, 58.2, 53.3, and 71.3% identical to those of HPIV4. An additional ORF, encoding 94 aa, was identified in the 475-nucleotide region between the M ORF and the F ORF. This ORF showed no homology to any proteins in the nonredundant database by BLASTP. Analysis of the transcriptional signals, however, failed to place the 94-aa-encoding ORF in the context of a transcriptional unit, suggesting that this gene is not expressed (6).

**FIG 1:**
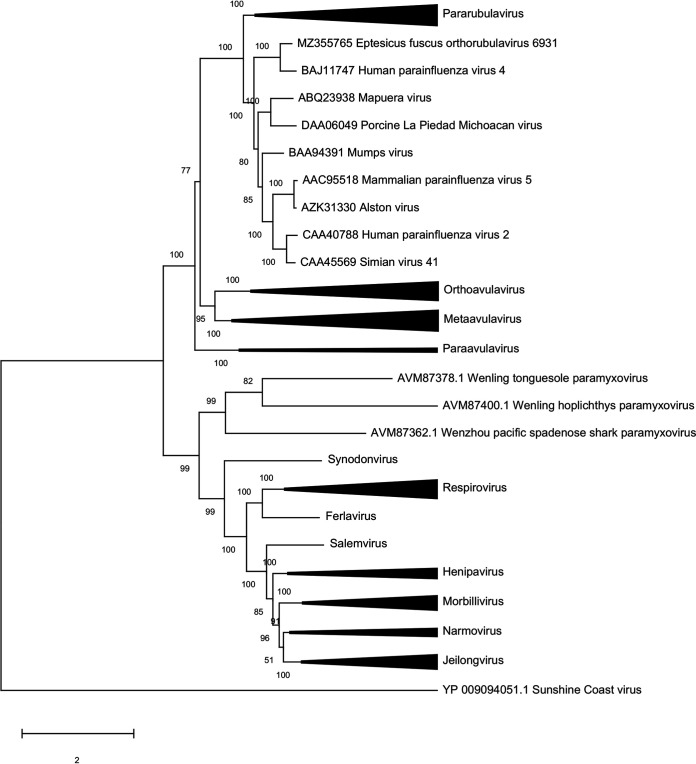
Phylogenetic analysis of the L protein sequences of representative paramyxoviruses. Sequences were aligned by ClustalW, with evolutionary histories inferred by maximum-likelihood analysis using the best-fitting LG+G+I model of evolution. Tree topology robustness was assessed with 500 bootstrap replicates.

Phylogenetic analyses were performed on complete L protein amino acid sequences downloaded from the ICTV *Paramyxoviridae* resources page (https://talk.ictvonline.org/ictv-reports/ictv_online_report/negative-sense-rna-viruses/w/paramyxoviridae/1197/resources-paramyxoviridae). Sequences were aligned by ClustalW, with phylogeny inferred by maximum-likelihood analysis using the best-fitting LG+G+I model of evolution; tree topology was assessed with 500 bootstrap replicates. EfORV occupied a sister clade closely related to that of HPIV4 (Fig. 1). The close phylogenetic relationship between EfORV and HPIV4 is similar to those seen for other bat and mammalian paramyxoviruses. The bat-originating Mapuera virus is genetically and antigenically related to the porcine orthorubulavirus La Piedad Michoacán virus, which causes blue-eye pig disease (7–9). Similarly, Alston orthorubulavirus, which was isolated from bats, is genetically and antigenically closely related to parainfluenza virus 5, which infects humans, monkeys, and dogs (10). The mumps orthorubulavirus species includes both human and bat mumps viruses, which are considered conspecific due to their high genetic, antigenic, and functional similarities (11).

Virus isolation from the tissue homogenate was attempted on Vero cells and immortalized big brown bat kidney cells without trypsin, under biosafety level 3 conditions. Virus isolation was also attempted on Vero 76 cells using medium containing trypsin. No cytopathic effects were apparent after two passages, and no virus growth was evident by PCR.

To assess the prevalence of EfORV, a TaqMan assay was designed based on the L gene sequence, as follows: forward, 5′-CCCATCAGAGTTCCCTACATTG; reverse, CCAGATATACACACCCGCTAATC; probe, 5′-6-carboxyfluorescein (FAM)-ATTGCTTCTCTTGCCCAAGTCCCA. Including the original sample, RNA was isolated from 90 bats that had been submitted for rabies testing. All bats were of the species Eptesicus fuscus; 84 originated from South Dakota, 5 from Minnesota, and 1 from Iowa. Eptesicus fuscus, commonly known as the big brown bat, is widespread throughout North America. The nonmigratory insectivorous bat commonly roosts in human structures, leading to frequent human exposure. The only positive sample was a submission in which EfORV was identified by sequencing (1/90 samples [1.1%]). South Dakota contains three distinct biomes, i.e., row crop and livestock agriculture in the eastern half of the state, with the western half being dominated by high plains grasslands and the coniferous Black Hills. The sample containing EfORV originated from the central high plains, from which our sample collection contained only two specimens. In addition to sampling biases, bat paramyxovirus shedding is periodic, with peak virus shedding occurring late in pregnancy and 2 months following parturition (12). The bat that was positive for EfORV was collected in April 2021, corresponding to gestation, although the sex was not determined.

HPIV4 is a significant human pathogen. In the United States from 2011 to 2019, 2,700,135 HPIV tests were conducted for patients with respiratory disease. Five percent of those tests were positive, with a subset of 13% being positive for HPIV4 (13). Similar to other parainfluenza viruses, HPIV4 was most frequently detected in children less than 2 years of age. A study in China similarly found the highest HPIV4 prevalence in children under 2 years of age, followed by a second peak among adults over 60 years of age (14). In Canada, HPIV4 infection was associated with bronchiolitis and pneumonia (15). The finding of a novel paramyxovirus in a bat with frequent human contact in a largely unsurveilled geographic region highlights the need for additional surveillance and underscores the necessity of pathogenesis studies.

### Data availability.

The genome of EfORV strain 6931 was submitted to GenBank under accession number MZ355765.
